# MRGPRX2 Activation by Rocuronium: Insights from Studies with Human Skin Mast Cells and Missense Variants

**DOI:** 10.3390/cells10010156

**Published:** 2021-01-15

**Authors:** Chalatip Chompunud Na Ayudhya, Aetas Amponnawarat, Saptarshi Roy, Carole A. Oskeritzian, Hydar Ali

**Affiliations:** 1 Department of Basic and Translational Sciences, University of Pennsylvania School of Dental Medicine, Philadelphia, PA 19104, USA; chalatip@upenn.edu (C.C.N.A.); aetasa@upenn.edu (A.A.); roysapta@upenn.edu (S.R.); 2 Department of Pathology, Microbiology and Immunology, University of South Carolina School of Medicine, Columbia, SC 20209, USA; Carole.Oskeritzian@uscmed.sc.edu

**Keywords:** anaphylaxis, mast cells, missense mutation, MrgprB2, MRGPRX2, rocuronium

## Abstract

Perioperative hypersensitivity (POH) to the neuromuscular blocking drug (NMBD) rocuronium was previously thought to be IgE and mast cell (MC)-mediated. However, the recent seminal observation that rocuronium induces degranulation in murine peritoneal MCs (PMCs) via Mas-related G protein-coupled receptor B2 (MrgprB2) led to the idea that POH to this drug involves the activation of MRGPRX2 (human ortholog of MrgprB2). Furthermore, based on the demonstration that a patient with POH to rocuronium displayed three missense mutations (M196I, L226P and L237P) in MRGPRX2’s transmembrane domains, it was proposed that this hypersensitivity reaction resulted from aberrant activation of this receptor. We found that rocuronium at 20 µg/mL caused degranulation in mouse PMCs via MrgprB2 but required at least 500 µg/mL to induce degranulation in human MCs via MRGPRX2. Furthermore, RBL-2H3 cells transiently expressing M196I, L226P and L237P variants did not display enhanced degranulation in response to rocuronium when compared to the wild-type receptor. These findings provide the first demonstration that rocuronium induces degranulation in human MCs via MRGPRX2. Furthermore, the important differences between MrgprB2 and MRGPRX2 and the inability of rocuronium to induce enhanced response in cells expressing MRGPRX2 variants suggest that the mechanism of its POH is more complex than previously thought.

## 1. Introduction

Anaphylactic reactions to drugs used during general anesthesia are rare, yet seriously life-threatening conditions that can occur with a mortality rate ranging from 3% to 9% [1]. Rocuronium is a widely used neuromuscular blocking drug (NMBD) that is frequently reported for perioperative hypersensitivity (POH) [2]. Recent evidence has shown that besides immunoglobulin E (IgE)/high-affinity IgE receptor (FcεRI)-dependent mast cell (MC) activation, rocuronium causes degranulation in murine peritoneal mast cells (PMCs) via the activation of a novel G protein-coupled receptor (GPCR) known as Mas-related GPCR-B2 (MrgprB2; human ortholog MRGPRX2) [3]. Additionally, some patients with rocuronium-induced POH displayed positive skin tests to irritating concentrations of rocuronium (1–10 mg/mL), despite demonstrating negative specific-IgE (sIgE) and/or basophil activation test (BAT) [4,5]. Based on these findings, it has been proposed that MRGPRX2 plays a critical role in rocuronium-induced POH in humans [6].

Despite one report showing that rocuronium induces degranulation in mouse PMCs via MrgprB2, evidence that it induces degranulation in human MCs via MRGPRX2 is lacking. McNeil et al. [3] showed that rocuronium causes intracellular Ca^2+^ mobilization in transfected HEK293 cells with EC_50_ values of 22.2 µg/mL and 261 µg/mL, for mouse MrgprB2 and human MRGPRX2, respectively. Although these authors showed that rocuronium induces robust degranulation in mouse PMCs via MrgprB2, its effect on human MCs was not reported [3]. More recent studies have shown that rocuronium, up to a concentration of 2 mg/mL, induces a small and transient Ca^2+^ mobilization in human peripheral CD34^+^ cell-derived MCs and a human MC line LAD2 cells via MRGPRX2 but this response does not provide a sufficient signal for degranulation [7,8,9]. The reason for the discrepancy between MrgprB2 and MRGPRX2 is not known but could reflect the fact that there is only ~53% sequence homology between these two receptors, resulting in different ligand-binding affinities [3,10]. Although high concentrations of rocuronium causes irritative skin reactions, the possibility that this is mediated via the activation of MRGPRX2 in human skin MCs has not been determined. 

Suzuki et al. [5] recently reported a patient who experienced severe POH to rocuronium. Intradermal injection of undiluted rocuronium (10 mg/mL) in this patient resulted in a positive skin reaction but a total IgE level was within normal limits and no sIgE to rocuronium was detected. Based on these findings, this POH reaction was diagnosed as rocuronium-induced non-IgE-mediated anaphylaxis. Sequence analysis of genomic DNA from the blood of this patient revealed the presence of three missense mutations (M196I, L226P and L237P) in MRGPRX2’s fifth and sixth transmembrane domains [5]. Rocuronium did not induce degranulation in rat basophilic leukemia (RBL-2H3) cells transiently expressing MRGPRX2 [5]. It was proposed that mutations found in MRGPRX2 of this patient with a rocuronium-induced reaction would render the receptor more susceptible to activation. However, this hypothesis has not been tested experimentally. 

The goals of the present study were to determine if rocuronium causes degranulation in mouse and human MCs and to test the hypothesis that missense mutations (M196I, L226P and L237P) in MRGPRX2’s transmembrane domains result in gain-of-function phenotype for MC degranulation by rocuronium. The data presented herein confirm the previous report that rocuronium induces degranulation in mouse PMCs via MrgprB2 but provide the first demonstration that it causes degranulation in human MCs via MRGPRX2, although requiring at least a 25-fold higher concentration to do so. Furthermore, functional studies with MRGPRX2 mutants found in a patient with rocuronium-induced POH suggest that the mechanism of its hypersensitivity is more complex than previously thought. 

## 2. Materials and Methods

### 2.1. Reagents

All cell culture reagents were purchased from Invitrogen (Carlsbad, CA, USA). Amaxa Nucleofector kit (Kit V) was obtained from Lonza (Gaithersburg, MD, USA). Recombinant mouse interleukin-3 (IL-3) and stem cell factor (SCF) and recombinant human SCF (rhSCF) were purchased from Peprotech (Rocky Hill, NJ, USA). Rocuronium bromide (CAS no. 119302-91-9, Item no. 23698) was purchased from Cayman Chemical (Ann Arbor, MI, USA). p-nitrophenyl-N-acetyl-β-D-glucosamine (PNAG) was from Sigma-Aldrich (St. Louis, MO, USA). Fluorescein isothiocyanate (FITC)-conjugated anti-human LAMP-1 and phycoerythrin (PE)-conjugated anti-human MRGPRX2 antibodies were from BioLegend (San Diego, CA, USA). Plasmid encoding hemagglutinin (HA)-tagged human MRGPRX2 in pReceiver-MO6 vector was obtained from GeneCopoeia (Rockville, MD, USA). 

### 2.2. Mice

C57BL/6 (wild-type; WT) mice were obtained from the Jackson Laboratory (Bar Harbor, ME, USA). MrgprB2^−/−^ mice in C57BL/6 background generated using CRISPR-Cas9-mediated gene deletion of *MrgprB2* were obtained from the CRISPR-Cas9 core facility of the University of Pennsylvania [11]. Mice were housed in pathogen-free cages on autoclaved hardwood bedding. Eight- to twelve-weeks-old male and female mice were used in this study. All experiments were approved by the Institutional Animal Care and Use Committee at The University of Pennsylvania. 

### 2.3. Cell Line Cultures

The human MC line LAD2 was provided by Dr. A. Kirshenbaum and Dr. D. Metcalfe (Laboratory of Allergic Diseases, National Institute of Allergy and Infectious Diseases, National Institutes of Health, Bethesda, MD, USA) and was maintained in complete StemPro-34 medium supplemented with l-glutamine (2 mM), penicillin (100 IU/mL), streptomycin (100 μg/mL), and rhSCF (100 ng/mL). Hemidepletions were performed weekly with media containing rhSCF [12].

RBL-2H3 cells were maintained as monolayer cultures in Dulbecco’s modified Eagle’s medium (DMEM) supplemented with 10% FBS, L-glutamine (2 mM), penicillin (100 IU/mL) and streptomycin (100 µg/mL) at 37 °C with 5% CO_2_ [13]. RBL-2H3 cells stably expressing MRGPRX2 (RBL-MRGPRX2) were used and maintained similarly in the presence of G-418 (1 mg/mL) [14,15].

### 2.4. Human Skin-Derived Mast Cell Isolation and Culture

Surgical skin samples were collected from the Cooperative Human Tissue Network of the National Cancer Institute, as approved by the Internal Review Board at the University of South Carolina. Skin MCs were harvested and cultured from 3 human donors as previously described [16]. Briefly, subcutaneous fat was removed by blunt dissection, and residual tissue was cut into 1 to 2 mm fragments and digested with type 2 collagenase (1.5 mg/mL), hyaluronidase (0.7 mg/mL), and type 1 DNase (0.3 mg/mL) in Hank’s Balanced Salt Solution (HBSS) for 2 h at 37 °C. The dispersed cells were collected by filtering through a No. 80 mesh sieve and resuspended in HBSS containing 1% fetal calf serum (FCS) and 10 mM HEPES. Cells were resuspended in HBSS and layered over 75% Percoll in a HBSS cushion and centrifuged at 700× *g* at room temperature for 20 min. Nucleated cells were collected from the buffer/Percoll interface. Percoll gradient-enriched cells were resuspended at a concentration of 1 × 10^6^ cells/mL in serum-free X-VIVO 15 medium containing 100 ng/mL rhSCF. MCs were used after 6–10 weeks of culture, when purity was nearly 100%, as confirmed with toluidine blue staining.

### 2.5. Peritoneal Mast Cell Isolation and Culture

PMCs were purified from WT and MrgprB2^−/−^ mice as described previously [17]. Briefly, the peritoneal cavity was lavaged with 10 mL of HBSS supplemented with 3% FCS and 10 mM HEPES. The cells were cultured in Iscove’s Modified Dulbecco’s Medium (IMDM) supplemented with 10% FCS, murine IL-3 (10 ng/mL), and murine SCF (30 ng/mL). After 48 h, non-adherent cells were removed and adherent cells were cultured in fresh medium for an additional 10–14 days. Suspension cells were used for experiments as PMCs. 

### 2.6. Mast Cell Staining

Toluidine blue and Alcian/Safranin staining were performed on the cultured PMCs (5 × 10^4^) as described previously [18]. Images were captured using a Nikon E600 microscope with a digital camera attached at 40× magnification and analyzed using Nikon NIS Elements software.

### 2.7. RNA Isolation and PCR 

RNA isolation and PCR was performed as described previously [17]. Briefly, total mRNA was isolated from WT and MrgprB2^−/−^ PMCs using the RNeasy Plus Mini Kit (Qiagen, Hilden, Germany) as per manufacturer’s instructions. cDNA was prepared using High Capacity cRNA Reverse Transcriptase Kit (Applied Biosystem, Foster City, CA, USA). PCR was performed using the MrgprB2 (Forward 5′-GTCACAGACCAGTTTAACACTTCC-3′ and Reverse 5′-CAGCCATAGCCAGGTTGAGAA-3′) and GAPDH (Forward 5′-CCATGACAACTTTGGCATTG-3′ and Reverse 5′-CCTGCTTCACCACCTTCTTG-3′) primers. PCR product was run in 2% agarose gel and image was acquired in iBright™ 1500 Imaging System (Thermo Scientific, Waltham, MA, USA).

### 2.8. Generation of RBL-2H3 Cells Transiently Expressing MRGPRX2 and Its Variants

Q5 site-directed mutagenesis kit (New England BioLabs, Ipswich, MA, USA) was used to generate MRGPRX2 variants in pReceiver-MO6 vector. To confirm the correct nucleotide sequences, each mutant was verified by DNA sequencing prior to transfection. The forward and reverse primers used for new variants are listed below.

M196I: Forward: 5′-TTTTATTCATCGTTCTCTGTGGGTCC-3′
Reverse: 5′-AAATCAGCCACGCTGCAG-3′;
L226P: Forward: 5′-CTGACCATCCCGCTCACAGTG-3′
Reverse: 5′-GTACAGCCTGGTCAGTGG-3′;
L237P: Forward: 5′-CTCTGCGGCCCGCCCTTTGGC-3′
Reverse: 5′-GAGGAACACCAGCACTGTGAGC-3′;

RBL-2H3 cells (2 × 10^6^) were transiently transfected with 2 µg of HA-tagged plasmid using the Amaxa Nucleofector Device and Amaxa Kit V according to the manufacturer’s protocol. Cells were used within 16–20 h after transfection.

### 2.9. MRGPRX2 Expression and Internalization Using Flow Cytometry

To induce MRGPRX2 internalization, cells (5 × 10^5^) were stimulated with rocuronium (2 mg/mL) for 30 min at 37 °C. To detect cell surface MRGPRX2 expression, cells were washed with FACS buffer (PBS containing 2% FCS and 0.02% sodium azide), incubated with the PE-conjugated anti-MRGPRX2 antibody for 30 min at 4 °C in the dark, and fixed in 1.5% paraformaldehyde. The samples were acquired in a BD LSR II flow cytometer (San Jose, CA, USA) and analyzed by WinList software. The adjusted mean fluorescent intensity (MFI) was calculated as MFI of sample/MFI of isotype control.

### 2.10. Degranulation Measured by β-Hexosaminidase Release Assay

RBL-2H3 cells (5 × 10^4^), LAD2 cells (1 × 10^4^), human skin-derived MCs (5 × 10^3^) and PMCs (1 × 10^4^) were seeded into a 96-well, white, clear-bottom cell culture plate in a total volume of 50 μL HEPES buffer containing 0.1% bovine serum albumin (BSA). Experimental groups were stimulated with different concentrations of rocuronium for 30 min at 37 °C. Cells without treatment were designated as controls. To determine the total β-hexosaminidase release, unstimulated cells were lysed in 50 μL of 0.1% Triton X-100. Aliquots (20 μL) of supernatants or cell lysates were incubated with 20 μL of 1 mM PNAG for 1 h at 37 °C. The reaction was stopped by adding 250 μL of stop buffer (0.1 M Na_2_CO_3_/0.1 M NaHCO_3_). The β-hexosaminidase release was assessed by measuring absorbance at 405 nm using Versamax microplate spectrophotometer (San Jose, CA, USA).

### 2.11. Degranulation Measured by the Surface Expression of Lysosomal-Associated Membrane Protein 1 (LAMP-1) 

Degranulation was also assessed by flow cytometric measurement of the surface expression of LAMP-1. Cells (5 × 10^5^) were stimulated with rocuronium (2 mg/mL) for 5 min, washed and exposed to FITC-conjugated anti-LAMP-1 antibody for 30 min at 4 °C. Cell surface expression of LAMP-1 was determined by flow cytometry as described above.

### 2.12. Statistical Analysis 

Data shown are mean ± standard error of the mean (SEM) values derived from at least three independent experiments. Statistical significance was determined using *t*-test and one- or two-way ANOVA. Differences were considered as statistically significant at a value * *p* < 0.05, ** *p* < 0.01, *** *p* < 0.001, and **** *p* < 0.0001. Data were analyzed by GraphPad Prism version 6.07.

## 3. Results

### 3.1. Rocuronium Activates Murine PMCs via MrgprB2

**Figure 1:**
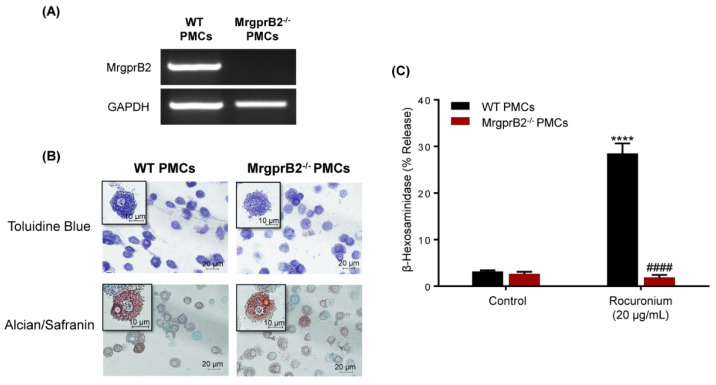
Rocuronium activates mouse peritoneal mast cells (PMCs) via MrgprB2. (**A**) PCR analysis was performed to determine the mRNA expression of MrgprB2 in wild-type (WT) and MrgprB2^−/−^ PMCs. GAPDH was used for normalization. (**B**) WT and MrgprB2^−/−^ PMCs were stained with Toluidine blue and Alcian/Safranin. The images were acquired at 40× resolution. Bars = 10 and 20 μm. (**C**) WT and MrgprB2^−/−^ PMCs were stimulated with rocuronium (20 μg/mL) for 30 min, and β-hexosaminidase release was determined. Data are expressed as mean ± SEM. Statistical significance was determined by two-way ANOVA with Tukey post-hoc test. **** *p* < 0.0001 compared to the control, #### *p* < 0.0001 compared between WT and MrgprB2^−/−^ groups.

McNeil et al. [3] showed that EC_50_ value for rocuronium-induced Ca^2+^ mobilization in HEK293 cells expressing MrgprB2 is 22.2 µg/mL. They also demonstrated that rocuronium at a concentration of 500 µg/mL induces substantial degranulation in mouse PMCs and that this response was abolished in cells obtained from MrgprB2^−/−^ mice [3]. Due to the recent reports that rocuronium, even at a concentration of 2 mg/mL, does not induce degranulation in human MCs [7,8,9], we first sought to validate the activity of rocuronium used in the present study by testing its ability to activate mouse MrgprB2. For this, we utilized MrgprB2^−/−^ mice generated by CRISPR-Cas9-mediated gene deletion and confirmed that the absence of the receptor transcript in PMCs (Figure 1A) had no effect on their differentiation and maturation as determined by Toluidine Blue and Alcian/Safranin staining (Figure 1B). Rocuronium, at concentration of 20 μg/mL, induced ~30% degranulation in PMCs obtained from the WT mice but this response was absent in cells obtained from MrgprB2^−/−^ mice (Figure 1C). It is noteworthy that McNeil et al. [3] showed that 500 µg/mL of rocuronium induced >80% degranulation in mouse PMCs. Thus, the data presented in Figure 1 are consistent with previously reported EC_50_ value for rocuronium-induced Ca^2+^ mobilization in transfected HEK293 cells and degranulation in mouse PMCs. 

### 3.2. Rocuronium Induces MRGPRX2-Mediated Degranulation in Human MCs

**Figure 2:**
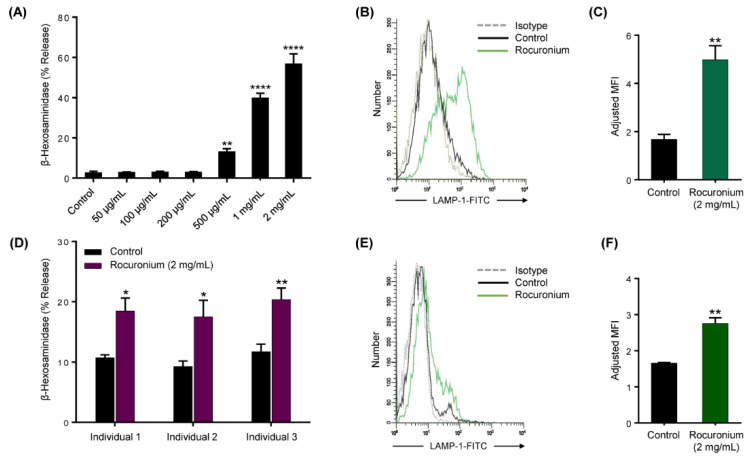
Rocuronium activates LAD2 cells and primary human skin mast cells (MCs) to cause degranulation. (**A**) LAD2 cells were exposed to buffer (control) or different concentrations of rocuronium for 30 min, and β-hexosaminidase release was determined. (**B**) Cells were stimulated with rocuronium (2 mg/mL) for 5 min, and LAMP-1 expression was determined by flow cytometry. Representative histograms of three independent experiments are shown. (**C**) The adjusted mean fluorescent intensity (MFI) levels of LAMP-1 expression are shown. Adjusted MFI was calculated as MFI of sample/MFI of isotype control. (**D**) Primary skin MCs were isolated and cultured from the human skin of 3 different donors. Skin-derived MCs were used to determine rocuronium-induced β-hexosaminidase release, and (**E**,**F**) LAMP-1 expression. All data points are the mean ± SEM of at least three experiments. For comparisons of two samples, a two-tailed unpaired *t*-test was used. For comparisons of multiple samples to a control group, one-way ANOVA with Dunnett’s post-hoc test was used. * *p* < 0.05, ** *p* < 0.01 and **** *p* < 0.0001.

Given that the EC_50_ for value for rocuronium-induced Ca^2+^ mobilization in HEK293 cells expressing MRGPRX2 is 261 µg/mL, we expected this concentration of the drug to induce degranulation in LAD2 cells naturally expressing the receptor [3]. However, we found that the lowest concentration of rocuronium that induced degranulation, as measured by β-hexosaminidase release, was 500 µg/mL and maximal response was obtained at 2 mg/mL (Figure 2A). We did not use rocuronium at concentrations higher than 2 mg/mL because of its reported cytotoxic effect at higher concentrations [7]. Rocuronium-induced degranulation in LAD2 cells was further confirmed by assessing the upregulation of LAMP-1 on the cell surface. Consistent with β-hexosaminidase release, rocuronium induced an increase in cell surface LAMP-1 expression on LAD2 cells as detected by flow cytometry (Figure 2B,C). To confirm the biological relevance of our studies with LAD2 cells, we cultured MCs isolated from the human skin of three different donors. We found that rocuronium (2 mg/mL) triggered degranulation as measured by β-hexosaminidase release and LAMP-1 expression (Figure 2D–F) but these responses were lower in magnitude than those observed with LAD2 cells.

**Figure 3:**
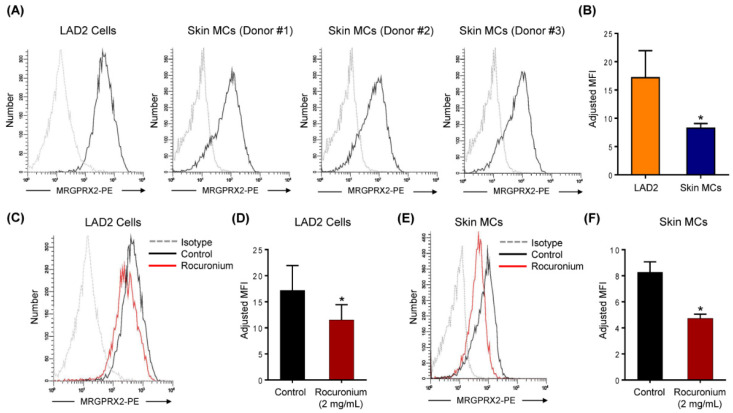
Rocuronium induces MRGPRX2 internalization in LAD2 cells and primary human skin MCs. (**A**) Flow cytometry histograms for MRGPRX2 cell surface expression (solid black line) and isotype (dotted gray line) are shown. Histograms are representative of three independent experiments. (**B**) The comparison of MRGPRX2 expression levels between human MC line LAD2 cells and primary human skin MCs. (**C**) LAD2 cells were stimulated with rocuronium (2 mg/mL) for 30 min, and MRGPRX2 internalization was determined by flow cytometry. (**D**) The adjusted MFI levels of MRGPRX2 cell surface expression are shown. (**E**,**F**) Rocuronium-induced MRGPRX2 internalization and adjusted MFI levels of MRGPRX2 cell surface expression were determined in primary human skin MCs. All data points are the mean ± SEM of at least three experiments. Statistical significance was determined by two-tailed unpaired *t*-test. * *p* < 0.05.

Cell surface expression of MRGPRX2 on LAD2 cells and primary skin MCs was determined by flow cytometry using PE-conjugated anti-MRGPRX2 antibody. As shown in Figure 3A,B, MRGPRX2 is expressed at a significantly lower level on skin MCs than LAD2 cells. This finding is consistent with the lower level of degranulation in skin MCs when compared to LAD2 cells (Figure 2). Codeine induces degranulation in human skin MCs via MRGPRX2 and it also causes receptor internalization as measured by loss of cell surface expression by flow cytometry [19]. To determine if rocuronium also causes MRGPRX2 internalization, LAD2 cells and skin MCs were exposed to rocuronium (2 mg/mL for 30 min) and cell surface receptor expression was determined by flow cytometry. As shown in Figure 3C–F, rocuronium caused significant loss of cell surface receptor expression in both LAD2 and skin MCs. These findings suggest that similar to codeine [19], rocuronium induces degranulation in human MCs via MRGPRX2 and causes receptor internalization.

### 3.3. MRGPRX2 Variants Presented in a Patient with POH to Rocuronium Do Not Display Gain-of-Function Phenotype for Rocuronium-Induced MC Degranulation

**Figure 4:**
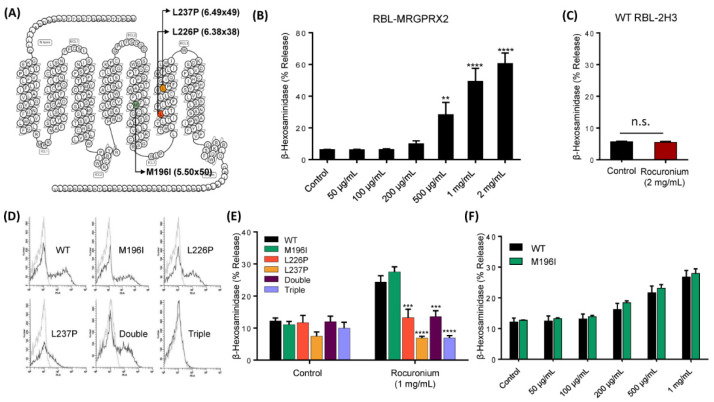
MRGPRX2 mutations rendered the receptor unresponsiveness to rocuronium. (**A**) Snake diagram of MRGPRX2 indicating three missense mutations identified in the patient. (**B**) RBL-MRGPRX2 were stimulated with different concentrations of rocuronium for 30 min, and β-hexosaminidase release was determined. (**C**) Untransfected WT RBL-2H3 cells were stimulated with rocuronium (2 mg/mL) for 30 min, and β-hexosaminidase release was determined. (**D**) Cell surface expression of WT MRGPRX2 and its variants was determined by flow cytometry. (**E**) Cells expressing WT MRGPRX2 and its variants were exposed to buffer (control) or rocuronium (1 mg/mL) for 30 min, and β-hexosaminidase release was determined. (**F**) Dose response of rocuronium-induced β-hexosaminidase release was determined in cells expressing WT MRGPRX2 and M196I. Data are the mean ± SEM of at least three experiments. For comparisons of two samples, two-tailed unpaired *t*-test was used. For comparisons of multiple samples to a control group, one-way ANOVA with Dunnett’s post-hoc test was used. ** *p* < 0.01, *** *p* < 0.001 and **** *p* < 0.0001.

Suzuki et al. [5] recently reported that a patient with severe rocuronium-induced POH harbors three missense mutations (M196I, L226P and L237P) in MRGPRX2’s fifth and sixth transmembrane domains (Figure 4A). We have previously utilized RBL-2H3 cells transiently expressing missense variants of MRGPRX2 to determine the impact of single nucleotide polymorphism on receptor function in response to a variety of agonists [20,21]. To assess if rocuronium induces degranulation in RBL-2H3 cells, we utilized cells stably expressing MRGPRX2 (RBL-MRGPRX2) [14,15]. We found that similar to the situation in LAD2 cells (Figure 2A), rocuronium caused dose-dependent degranulation (500 µg/mL–2 mg/mL) in RBL-MRGPRX2 but not in untransfected cells (Figure 4B,C). 

To test the possibility that MRGPRX2 mutations reported in this patient render the receptor more susceptible to rocuronium-induced MC degranulation, we first constructed cDNAs encoding MRGPRX2 mutants—M196I, L226P and L237P—and generated separate transient transfectants expressing each variant in RBL-2H3 cells. Flow cytometry analysis demonstrated that while M196I and L226P variants were expressed on the cell surface at a level similar to WT MRGPRX2, L237P variant displayed reduced expression (Figure 4D). Surprisingly, we found that cells expressing L226P and L237P variants showed loss-of-function phenotype for MC degranulation in response to rocuronium, while cells expressing M196I variant responded similarly to the WT receptor (Figure 4E). Rocuronium at the concentration of 1 mg/mL evoked nearly peak response in cells expressing WT-MRGPRX2 (Figure 4B), thus it would not be able to identify variants with enhanced activity. To further validate if M196I variant possibly displays a gain-of-function phenotype by rendering the mutated receptor more responsive at lower concentrations of the drug, we performed a dose response of rocuronium-induced degranulation in cells expressing M196I variant and the WT receptor. We found that rocuronium at different concentrations induces similar degranulation in cells expressing M196I variant and the WT receptor.

It is possible that the patient who displayed rocuronium-induced POH harbors more than one mutation in the same MRGPRX2 allele [5]. Because we do not have any information on the MRGPRX2 status of the patient’s parents, we are unable to determine that possibility. However, we constructed cDNAs encoding double (M196I, L226P) and triple (M196I, L226P, L237P) variants, generated transient transfectants in RBL-2H3 cells and determined cell surface expression by flow cytometry. We found that the double mutant was expressed on the surface of RBL-2H3 cells but the triple mutant was not (Figure 4D). However, both double and triple mutants were resistant to rocuronium-induced degranulation (Figure 4E). Thus, the demonstration that an individual with severe rocuronium-induced POH harbors MRGPRX2 mutations that renders loss-of-function phenotype for MC degranulation does not support the hypothesis that MRGPRX2 participates in rocuronium hypersensitivity in this patient.

## 4. Discussion

The seminal observation that rocuronium activates murine PMCs via MrgprB2 [3] led to the idea that POH to this drug involves the activation of MRGPRX2. However, attempts to demonstrate rocuronium-induced degranulation in MRGPRX2-expressing human MCs have not been successful to date [7,8,9]. A number of patients with rocuronium-induced POH displayed positive intradermal skin test (IDT) to rocuronium at irritative concentrations (1 mg/mL and 10 mg/mL), but were negative for both sIgE and BAT [4,5]. These individuals were diagnosed with non-IgE-mediated anaphylaxis and it was proposed that MRGPRX2 plays a critical role in rocuronium-induced POH without any evidence of MC degranulation by MRGPRX2. The novel findings of the present study are that rocuronium induces degranulation in human and murine MCs with different efficacies, and that missense mutations found in MRGPRX2 of a patient with rocuronium hypersensitivity do not render the receptor more susceptible to activation. These findings suggest that mechanism of rocuronium-induced POH is more complex than previously thought. 

The data presented herein provide the first demonstration that irritating concentrations of rocuronium that were reported to induce skin reactions in patients with drug hypersensitivity [4,5] induce degranulation in LAD2 cells, MRGPRX2-expressing RBL-2H3 cells and primary human skin MCs. The reason for the difference between the present study and previous reports is not clear but could reflect differences in receptor expression on different cell types and concentrations of rocuronium used. For example, Fernandopulle et al. [8] showed that while 200 µM rocuronium (~120 µg/mL) induces a small but significant Ca^2+^ response, this was not associated with degranulation in LAD2 cells. The demonstration in the present study that this concentration of rocuronium does not induce degranulation in LAD2 and transfected RBL-2H3 cells is consistent with previous report and suggest that small increase in Ca^2+^ mobilization does not provide a sufficient signal for MC degranulation. 

We found that although rocuronium caused degranulation in human skin MCs, the magnitude of the response was lower than that observed in LAD2 cells and transfected RBL-2H3 cells and this difference is associated with differences in cell surface receptor expression. Elst et al. [7] developed an MRGPRX2 knockdown strategy in primary human peripheral CD34^+^ cell-derived MCs to study MRGPRX2-mediated drug reactions in vitro. These MCs consist of almost equal numbers of MRGPRX2^+^ and MRGPRX2^−^ cells. The authors reported that a high non-toxic concentration of rocuronium (1640 µM; ~1 mg/mL) induced a small but transient Ca^2+^ mobilization via MRGPRX2 but this response was not associated with MC degranulation [7]. This probably reflects a low level of receptor expression, which provides a sufficient signal for a small and transient Ca^2+^ mobilization but not for degranulation. The report by Navinés-Ferrer et al. [9] that rocuronium at a concentration of 2 mg/mL does not induce degranulation in LAD2 cells is inconsistent with our finding in this study and is difficult to explain. One possibility is that these authors utilized an inactive preparation of the drug as they did not perform control experiments to validate its activity using mouse PMCs or HEK293 cells expressing MRGPRX2 or MrgprB2. 

The transfection and functional studies performed in this study were based on MRGPRX2 mutations present in one patient with rocuronium-induced POH. The hypothesis that these mutations would render the receptor more responsive to rocuronium was not realized [5]. To more clearly define the roles of these MRGPRX2 mutations on the responsiveness to rocuronium, it will be important to generate MCs from CD34^+^ cells of this individual and to compare the expression of MRGPRX2 and their responsiveness to rocuronium with MCs derived from the CD34^+^ cells of normal individuals. For this patient, the diagnosis of rocuronium-induced POH was made based on a positive IDT with undiluted rocuronium (10 mg/mL). However, in the present study, we demonstrated that rocuronium at a concentration of 2 mg/mL induces degranulation in normal human skin MCs. These findings suggest that skin reactions induced by irritative concentrations of rocuronium are a normal part of skin MC activation via MRGPRX2 and are unrelated to hypersensitivity. Furthermore, a negative rocuronium sIgE result does not rule out an IgE-mediated response to rocuronium or cross-reactivity with other drugs used in this patient [22,23]. 

It is, however, possible that other mutations in MRGPRX2 can render the receptor more susceptible to activation by rocuronium and other drugs, making it an important target for hypersensitivity reactions. Examination of the GPCRdb database (GPCRdb.org) [24] reveals the presence of 72 missense MRGPRX2 variants with possible deleterious effects. It is therefore possible that one or more of these or other mutations positively modulate MRGPRX2 expression, ligand-binding affinity and signaling to promote hypersensitivity in the absence of IgE [21,25]. Of interest, Lansu et al. [26] identified an MRGPRX2 mutation in its ligand-binding pocket (E164D) that increases the receptor affinity for drugs used in anesthesia. Sequence alignment predicts that MrgprB2’s E171 is likely the residue that “sits” in the MRGPRX2 E164 position [27]. Taken together, these findings suggest that individuals harboring missense mutations in MRGPRX2’s ligand-binding pocket that makes the receptor function similar to MrgprB2 may display rocuronium-induced POH via MRGPRX2. Therefore, analysis of MRGPRX2 polymorphisms in the genome of suspected patients together with studies to determine their expression level and susceptibility to degranulation may enable us to determine specific MRGPRX2 variants that are involved in POH to rocuronium and other drugs used during general anesthesia.

## 5. Conclusions

In summary, the present study demonstrated that rocuronium induces degranulation in murine and human MCs via MrgprB2 and MRGPRX2, respectively, but with different affinities, indicating important functional differences between these receptors. MRGPRX2 mutations recently reported in a patient with POH to rocuronium displayed loss-of-function phenotype, thus disputing the role of MRGPRX2 in rocuronium-induced POH and suggesting that the mechanism of rocuronium-induced POH is more complex than previously thought.

## Data Availability

No datasets were generated or analyzed during this study.
